# Adiponectin is Protective against Oxidative Stress Induced Cytotoxicity in Amyloid-Beta Neurotoxicity

**DOI:** 10.1371/journal.pone.0052354

**Published:** 2012-12-27

**Authors:** Koon-Ho Chan, Karen Siu-Ling Lam, On-Yin Cheng, Jason Shing-Cheong Kwan, Philip Wing-Lok Ho, Kenneth King-Yip Cheng, Sookja Kim Chung, Jessica Wing-Man Ho, Vivian Yawei Guo, Almin Xu

**Affiliations:** 1 University Department of Medicine, LKS Faculty of Medicine, The University of Hong Kong, Hong Kong, Special Administrative Region, China; 2 Research Center of Heart, Brain, Hormone and Healthy Aging, LKS Faculty of Medicine, The University of Hong Kong, Hong Kong, Special Administrative Region, China; 3 Hong Kong University Alzheimer’s Disease Research Network, LKS Faculty of Medicine, The University of Hong Kong, Hong Kong, Special Administrative Region, China; 4 Neuroimmunology and Neuroinflammation Research Laboratory, LKS Faculty of Medicine, The University of Hong Kong, Hong Kong, Special Administrative Region, China; Federal University of Rio de Janeiro, Brazil

## Abstract

Beta-amyloid (Aβ ) neurotoxicity is important in Alzheimer’s disease (AD) pathogenesis. Aβ neurotoxicity causes oxidative stress, inflammation and mitochondrial damage resulting in neuronal degeneration and death. Oxidative stress, inflammation and mitochondrial failure are also pathophysiological mechanisms of type 2 diabetes (T_2_DM) which is characterized by insulin resistance. Interestingly, T_2_DM increases risk to develop AD which is associated with reduced neuronal insulin sensitivity (central insulin resistance). We studied the potential protective effect of adiponectin (an adipokine with insulin-sensitizing, anti-inflammatory and anti-oxidant properties) against Aβ neurotoxicity in human neuroblastoma cells (SH-SY5Y) transfected with the Swedish amyloid precursor protein (Sw-APP) mutant, which overproduced Aβ with abnormal intracellular Aβ accumulation. Cytotoxicity was measured by assay for lactate dehydrogenase (LDH) released upon cell death and lysis. Our results revealed that Sw-APP transfected SH-SY5Y cells expressed both adiponectin receptor 1 and 2, and had increased AMP-activated protein kinase (AMPK) activation and enhanced nuclear factor-kappa B (NF-κB) activation compared to control empty-vector transfected SH-SY5Y cells. Importantly, adiponectin at physiological concentration of 10 µg/ml protected Sw-APP transfected SH-SY5Y cells against cytotoxicity under oxidative stress induced by hydrogen peroxide. This neuroprotective action of adiponectin against Aβ neurotoxicity-induced cytotoxicity under oxidative stress involved 1) AMPK activation mediated via the endosomal adaptor protein APPL1 (adaptor protein with phosphotyrosine binding, pleckstrin homology domains and leucine zipper motif) and possibly 2) suppression of NF-κB activation. This raises the possibility of novel therapies for AD such as adiponectin receptor agonists.

## Introduction

Alzheimer’s disease (AD) is the most common cause of dementia in the elderly with significant morbidity and mortality [1]. The exact pathogenetic mechanisms underlyng AD are uncertain. One extensively studied mechanism is neurotoxicity mediated by beta-amyloid (Aβ) [2]–[5]. Histopathological studies of brain from AD patient reveal extracellular accumulation of senile plaques containing Aβ fibrils, intracellular accumulation of neurofibrillary tangles containing hyperphosphorylated tau, neuronal loss, amyloid angiopathy, and inflammation [1], [5]–[6]. Aβ peptides, predominantly Aβ40 and Aβ42, are derived from cleavage of amyloid precursor protein (APP), by β secretase and γ secretase [2]. Aβ exist in different forms including monomers (peptides), oligomers, protofibrils and fibrils [7]. The pathogenetic role of Aβ in AD is strongly supported by the observation that familial AD patients have mutations affecting proteins involved in Aβ production or processing such as APP, presenilin1 and presenilin 2. An example is the Swedish APP mutation (Sw-APP, APP_K670N, M671L_ ) that causes familial early-onset AD [8]. Aβ is neurotoxic [2]–[4]. Recent evidences suggest that Aβ oligomers are directly toxic to neurons and play important roles in early AD [9]–[12]. Aβ oligomers inhibit long-term potentiation in hippocampal neurons [12], impair neuronal synaptic transmission by causing loss of excitatory synapses and dendritic spines [13]–[14], and may induce uncontrolled ion flux by forming Ca^2+^-permeable pores in the lipid membrane [15]–[16].

Type 2 diabetes mellitus (T_2_DM) is, similar to AD, common in the elderly with significant morbidity and mortality. Interestingly, several pathophysiological features of T_2_DM are also found in AD. These include 1) insulin resistance, 2) inflammation, 3) oxidative stress, and 4) aberrant lipid metabolism [17]. In AD, there are 1) central insulin resistance resulting from reduction of insulin receptors and desensitization of insulin receptors in neurons [18]–[21], 2) Aβ induced microglial and astrocytic activation and release of inflammatory mediators which lead to neuroinflammation [22]–[24], 3) inhibition of enzymes for mitochondrial oxidative phosphorylation by Aβ leads to increased production of reactive oxygen species (ROS) which cause oxidative stress [25]–[26], and 4) the risk of apolipoprotein E (ApoE) ε4 allele. The Rotterdam study reported that T_2_DM doubled the risk of dementia and patients on insulin had 4 times the risk, suggesting that T_2_DM increases the risk to develop AD [27]. Consistently, T_2_DM patients have elevated serum levels of pro-inflammatory cytokines including IL-1, IL-6 and TNFα and display increased risk of cognitive decline than those without T_2_DM [28]–[29]. The term type 3 diabetes is proposed for AD [21], [30]. Takeda et al. crossed APP23 transgenic mice expressing Sw-APP mutant (mouse AD model) with leptin-deficient ob/ob mice (mouse DM model) and observed that onset of diabetes exacerbated AD-like cognitive dysfunction without increase in brain Aβ burden in the double transgenic mice (Sw-APP ob/ob) mice. Remarkably, the Sw-APP ob/ob mice had cerebrovascular inflammation and severe amyloid angiopathy. The investigators concluded that diabetes accelerated memory dysfunction via inflammation and Aβ deposition in cerebrovasculature [31].

Adiponectin is a serum adipokine secreted predominantly by adipocytes and possesses insulin-sensitizing, anti-inflammatory and anti-oxidant properties [32]–[33]. Serum adiponectin level is decreased in obesity, obesity-related insulin resistance, T_2_DM [34]–[35] and chronic inflammatory diseases such as coronary artery disease [36]. Adiponectin is protective against many major obesity-related pathologies including hypertension, atherosclerosis, non-alcoholic fatty liver, non-alcoholic steatohepatitis, heart failure, airway inflammation and some cancers [37]. Adiponectin acts by binding to its receptors, adiponectin receptor type 1 (AdipoR1) and type 2 (AdipoR2). Most physiological actions of adiponectin are mediated by activation of AMP-activated protein kinase (AMPK) via phosphorylation at threonine^172^ (Thr^172^) to yield phosphorylated AMPK (pAMPK). This adiponectin signaling pathway requires an endosomal adaptor protein, APPL1 (adaptor protein with phosphotyrosine binding, pleckstrin homology domains and leucine zipper motif) [38]. We hypothesize that adiponectin is protective against Aβ neurotoxicity in AD. In this communication, we report that adiponectin is protective against oxidative stress-induced cytotoxicity in human neuroblastoma cells under Aβ neurotoxicity and its underlying mechanisms of neuroprotection.

## Methods

The study was approved by the Hong Kong University-Hong Kong West Cluster Institutional Review Board. Human neuroblastoma cells (SH-SY5Y cell line) were transfected with the wild-type human APP (wt-APP) or Swedish-APP mutant (Sw-APP), and control being SH-SY5Y cells transfected with empty-vector. Cultured SH-SY5Y cells transfected with Sw-APP mutant overexpress APP with increased Aβ production by 5–6 fold, concomitant increase in secreted Aβ42 and Aβ40 and abnormal intracellular Aβ accumulation [39]–[41]. Cells transfected with wt-APP overexpress APP with increased Aβ production, but without abnormal intracellular Aβ accumulation [42]. The processing pathways for secreted Aβ and intracellular Aβ are different [41], [43]. SH-SY5Y cells transfected with Sw-APP mutant were used as an in-vitro system for further study. PC-12 (rat pheochromocytoma) cells transfected with Sw-APP mutant were shown to have increased Aβ production and enhanced vulnerability to oxidative stress-induced apoptosis [44].

### Site-directed Mutagenesis of the Sw-APP

Site-directed mutagenesis was performed by polymerase chain reaction (PCR) using the QuickChange Lightning Site-directed Mutagenesis Kit (Stratagene) according to the manufacturer’s protocol. The mutagenic primers used for generating the Sw-APP-mutated construct were: forward GGAGATCTCTGAAGTGAACTTGGATGCAGAATTCCGAC, and reverse GTCGGAATTCTGCATCCAAGTTCACTTCAGAGATCTCC. Non-mutated DNA was removed by DpnI endonucleases digestion, and vector DNA containing the desired mutations was transformed into XL10-Gold Ultracompetent cells (Stratagene). The presence of the mutated sequence was confirmed by sequencing both strands of the construct.

### Cell Culture and Transfection

SH-SY5Y cell line was obtained from ATCC and maintained at 37°C (5% CO_2_/95% air) in DMEM containing 10% fetal bovine serum (FBS) (Invitrogen, USA) and 1% penicillin and streptomycin. SH-SY5Y cells were transfected with empty-vector, vector carrying wt-APP and Sw-APP mutant under standard protocols using Lipofectamine 2000 (Invitrogen, USA) according to manufacturer’s instructions. Transfection was confirmed with DNA sequencing and stably transfected cells were selected with G418. Expression of the Sw-APP mutant with abnormal increase of intracellular accumulation of Aβ was confirmed with western blot of cell lysates using rabbit antibody against amino acids 1–16 of the amino terminal of human Aβ (Santa Cruz, CA).

### Reverse Transcriptase Polymerase Chain Reaction for Expression of Adiponectin Receptors

Reverse transcriptase-polymerase chain reaction (RT-PCR) was performed to study the expression of adiponectin receptors, AdipoR1 and AdipoR2, by SH-SY5Y cells. The primers used for human AdipoR1 are: forward GAGCATCTTCCGCATTCATA and reverse AAGAGCCAGGAGAAGCTGAG. The primers used for human AdipoR2 are: forward GACTTCCTCTTGCATGGACA and reverse AAAGGAGATATTTGGGCGAA.

### Enzyme Linked Immunosorbent Assay (ELISA) for Aβ Oligomers

Concentrations of secreted Aβ oligomers in culture medium of the three groups of cells were measured using ELISA recently reported by our group [45] and compared using ANOVA. In brief, the ELISA employed rabbit antibody against Aβ N-terminal (Aβ residues1–14) (Abcam) as the capturing antibody and the Aβ oligomers antibody, 7A1a (New England Agent), as the detecting antibody.

### Measurement of Cytotoxicity under Oxidative Stress and Neuroprotection by Adiponectin

Cell death and lysis (cytotoxicity) was assessed by examining under light microscope and measured by assay of lactate dehydrogenase (LDH) level. Cells were examined under light microscope at 10X and 100X for cell morphology with loss of dendritic spines (ballooning) as features suggestive of cell death. LDH release into medium was quantitated by cytotoxicity detection kit (Roche Applied Science, Germany) to assess cytotoxicity of the three groups of SH-SY5Y cells: 1) cells transfected with empty-vector, 2) cells transfected with wt-APP and 3) cells transfected with Sw-APP mutant before and after exposure to oxidative stress. Oxidative stress was induced with addition of H_2_O_2_ to culture medium at 200 µM, 400 µM and 800 µM for 2 hours. Sw-APP transfected SH-SY5Y cells (2×10^5^) were seeded on each well of a 24-well plate one day before use, each well contained 500 µl of DMEM containing 10% FBS (Invitrogen, USA) and 1% penicillin and streptomycin. Two hours before addition of H_2_O_2_, fresh culture medium without FBS was replaced and LDH level was measured 2 hours after addition of H_2_O_2_ according to manufacturer’s instructions. Potential neuroprotective effect of adiponectin was studied using Sw-APP transfected SH-SY5Y cells by adding adiponectin to fresh culture medium without FBS at 10 µg/ml for 2 hours before addition of H_2_O_2_. Mouse recombinant adiponectin was produced and purified as described previously [46]. LDH levels were used to calculate percentage cytotoxicity which was expressed as fold of cytotoxicity compared to percentage cytotoxicity of cells without adiponectin treatment or exposure to H_2_O_2_. Folds of cytotoxicity was compared between cells without adiponectin treatment or exposure to H_2_O_2_ (fold = 1), cells without adiponectin treatment before exposure to H_2_O_2_ and cells pretreated with adiponectin before exposure to H_2_O_2._

### Immunoblotting

Culture medium was aspirated from wells of 24-well plate. Cells were washed with ice-cold 1×PBS and removed. 100 µl of 1x lysis buffer (Cell Signaling Technology, USA) was added to each well and incubated on ice for 20 minutes with gentle shaking to allow complete lysis. The cell lysate was transferred to a 1.5 ml centrifuge tube and centrifuged at 14,000 rpm at 4°C for 10 minutes. After centrifugation, the supernatant was transferred to a new 1.5 ml centrifuge tube for Western blot analysis. Lysates of cultured cells were assessed for Aβ oligomers, by standard SDS-PAGE electrophoresis and immunoblotting using an Aβ oligomer antibody, 7A1a, as described recently [45]. In addition, expression levels of APPL1, total AMPK and pAMPK were studied by standard SDS-PAGE and immunoblotting using anti-APPL1 antibody and anti-pAMPK antibody as primary antibodies. Anti-human APPL1 antibody was synthezed by immunization of New Zealand female rabbits with the recombinant full-length APPL1 produced from Escherichia Coli using protocol described previously [47]. Antibody against AMPKα subunit phosphorylated at threonine^172^ (anti-pAMPK Thr^172^) was purchased from Cell Signaling (Beverly, MA, USA). The specific signals were amplified by addition of horseradish peroxidase-conjugated antibodies and visualized using an enhanced chemiluminescence (Amersham, UK).

### Knockdown of AdipoR1, AdipoR2 and APPL1 Expression by siRNA

Knockdown of AdipoR1 and AdipoR2 expression in Sw-APP transfected SH-SY5Y cells was performed using RNA interference by transfection with duplex stealth interference RNA (siRNA) for adipoR1 and adipoR2 (Invitrogen, USA) or scrambled RNA (negative control) using Lipofectamine 2000 according to manufacturer’s instructions as previously described by our group [47]. Knockdown of APPL1 expression in Sw-APP transfected SH-SY5Y cells was achieved similarly by siRNA for APPL1 (Invitrogen, USA) or scrambled RNA (negative control). Successful knockdown of AdipoR1, AdipoR2 and APPL1 expression was confirmed by western blot of cell lysates. Cells were then pretreated with adiponectin at 10 µg/ml for 2 hours before addition of H_2_O_2_ (400 µM) followed by cytotoxicity assay 2 hours later to study whether the neuroprotective effect of adiponectin required expression of AdipoR1, AdipoR2 and APPL1.

### Western Blot for NF-κB in Nuclear Fraction of SH-SY5Y Cells

Levels of NF-κB activation of the three groups of cells, empty-vector transfected, wt-APP transfected and Sw-APP transfected SH-SY5Y cells were studied by western blot for NF-κB p65 in nuclear fraction of the cells. Nuclear extracts of the SH-SY5Y cells were prepared according to the standard procedures of a Nuclear Extraction Kit (Panomics, Inc.; Beijing, China). Protein concentration of the nuclear extracts was measured, followed by standard procedures of protein transfer of western blot. The nitrocellulose membrane was incubated with NF-κB p65 antibody (Santa Cruz, California; dilution 1∶1,000) or histone H3 antibody (Abcam, Inc., U.K.; dilution 1∶1,000) as primary antibody at 4°C for overnight. After washing with 1X TBST buffer 3 times (5 min each), the membranes were incubated with anti-rabbit secondary antibody (DAKO, Denmark; dilution 1∶5,000) at RT for one hour and then washed again with 1X TBST buffer 3 times. The membranes were then immersed in ECL detection solution (Amersham Biosciences) for 1 min in darkness. Excess detection solution was drained off and the membrane was then wrapped in transparent wrapping film and exposed to X-ray film for 2 min. Images of the X-ray film were analyzed with Image-J. The effect of adiponectin on NF-κB activation of Sw-APP transfected SH-SY5Y cells was further studied. Sw-APP transfected SH-SY5Y cells were cultured in 6-well plate at one million cells per well one night before the experiment. Sw-APP transfected SH-SY5Y cells were divided into 4 groups: control cells without pretreatment with adiponectin (ADN) or exposure to H_2_O_2_ (group 1), cells treated with ADN (10 µg/ml) for 2 hours without exposure to H_2_O_2_ (group 2), cells exposed to 400 µM H_2_O_2_ for 2 hours without ADN pretreatment before exposure to H_2_O_2_ (group 3), and cells pretreated with ADN for 2 hours before exposure to 400 µM H_2_O_2_ for 2 hours (group 4). After treatment, nuclear extracts of the cells were prepared and western blot for NF-κB p65 was performed as described above. The amount of NF-κB p65 was quantitated by Image-J analysis.

### Statistical Analysis

Western blot results of different groups of cells were quantified by Image-J and compared by one-way ANOVA using Newman-Keuls multiple comparison test with p-value <0.05 considered as statistically significant. All ELISA and cytotoxicity experiments were performed at least 3 times and the mean values of results from different groups of cells were similarly compared by one-way ANOVA using Newman-Keuls multiple comparison test with p-value <0.05 considered as statistically significant.

## Results

### Human Neuroblastoma Cells Express both Adiponectin Receptor 1 and 2

**Figure 1:**
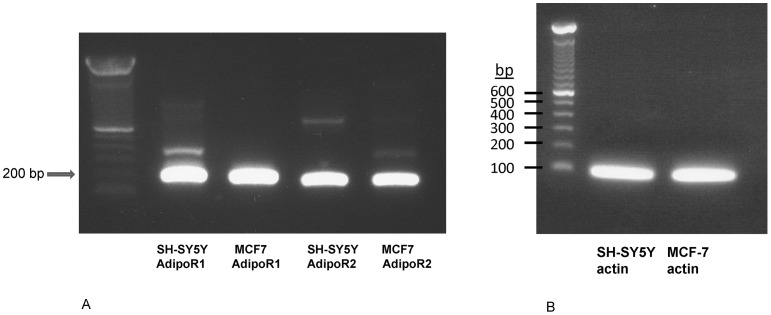
Human neuroblastoma cells (SH-SY5Ycells) express both adiponectin receptor 1 and 2. **A.** Reverse transcriptase polymerase chain reaction (RT-PCR) for expression of adiponectin receptor 1 (AdipoR1) and 2 (AdipoR2) in human neuroblastoma cells (SH-SY5Ycells). RT-PCR revealed the presence of PCR products of ∼200 base pair (bp) corresponding to the presence of messenger RNA for AdipoR 1 and 2. MCF7 cells serve as the positive control cells which express both AdipoR 1 and AdipoR 2. **B.** Actin serve as the housekeeping gene and is expressed by both SH-SY5Y and MCF-7 cells (PCR product of 78 bp). Experiments were performed twice with consistent results.

RT-PCR results demonstrated that SH-SY5Y cells express both AdipoR1 and AdipoR2 (Figure 1). A previous study has demonstrated that SH-SY5Y cells express AdipoR2 by western blot analysis [48].

### Human Neuroblastoma Cells Stably Transfected with Sw-APP Mutant and wt-APP had Increased AMPK Activation

**Figure 2:**
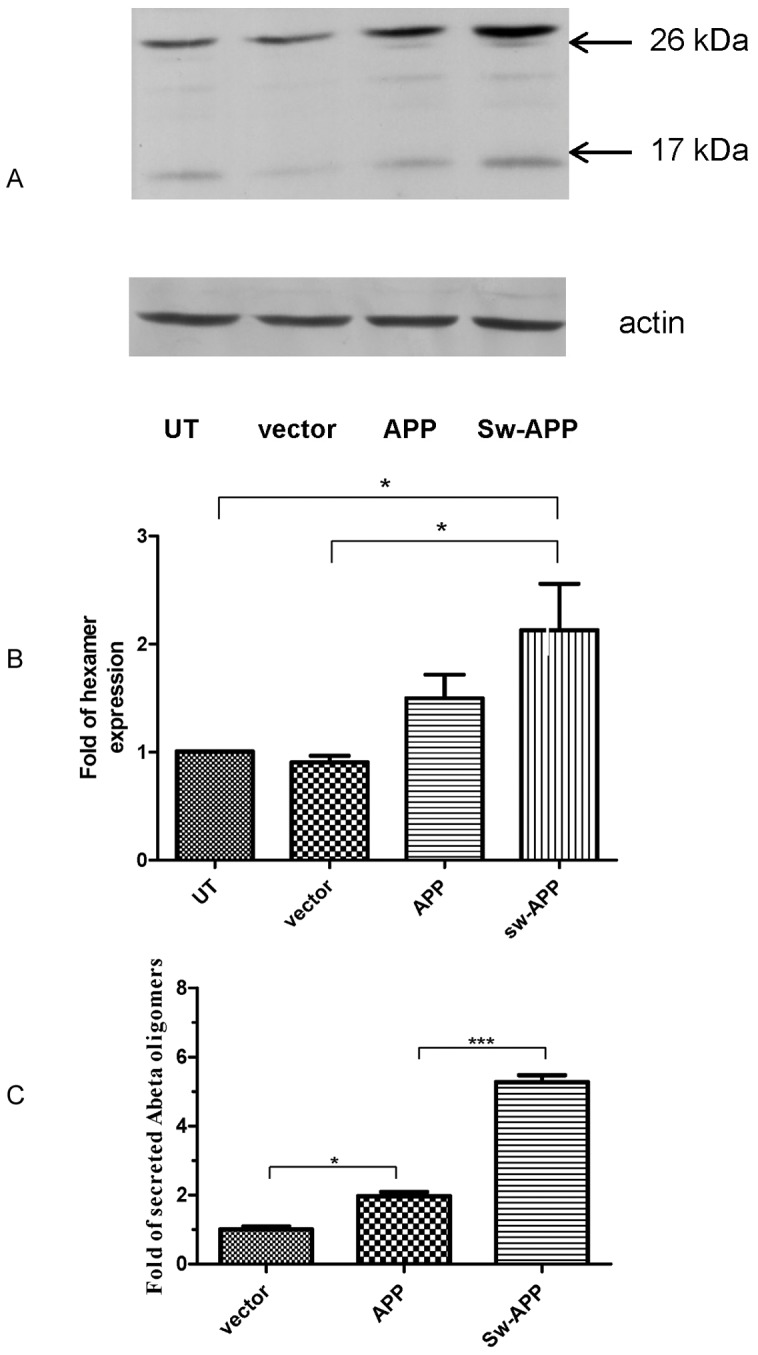
Sw-APP transfected SH-SY5Y cells secrete Aβ oligomers and had increased intracellular Aβ oligomers level. **A.** Western blot analysis of cell lysates with Aβ antibody. Immunoblotting demonstrated that SH-SY5Y cells transfected with Swedish amyloid precursor protein (Sw-APP) mutant had increased amount of intracellular Aβ oligomers (tetramers and hexamers) compared to SH-SY5Y cells transfected with wild-type amyloid precursor protein (APP), empty-vector (vector) or untransfected SH-SY5Y cells (UT). **B.** Image J quantitiative analysis confirmed that Sw-APP transfected cells (Sw-APP) had increased amount of intracellular hexamers compared to empty-vector transfected (vector) (p<0.05) or untransfected (UT) cells (p<0.05). **C.** ELISA for concentration of secreted Aβ oligomers in cultured medium. Sw-APP transfected SH-SY5Y cells (Sw-APP) secreted greater amount of extracellular Aβ oligomers than wt-APP transfected cells (APP) (p = 0.0001), which also secreted greater amount of extracellular Aβ oligomers than empty-vector transfected cells (vector) (p<0.05). Results shown were mean values from 3 independent cultures, and compared by one-way ANOVA using Newman-Keuls multiple comparison test.

**Figure 3:**
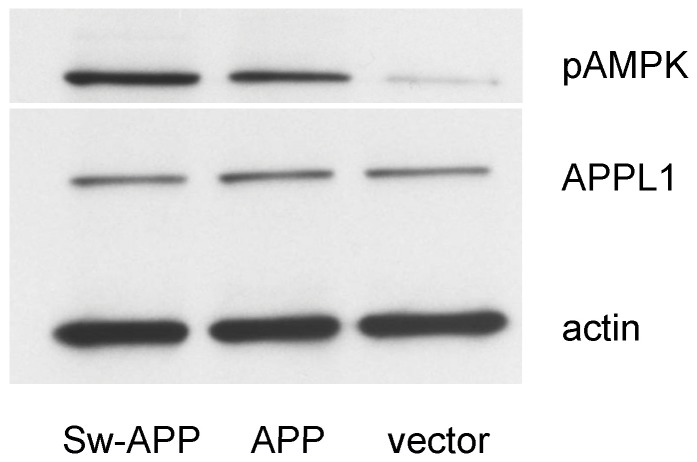
Western blot analysis of cell lysates for phosphorylated AMP-activated protein kinase (pAMPK) and the endosomal adaptor protein APPL-1 (adaptor protein with phosphotyrosine binding, pleckstrin homology domains and leucine zipper motif). Immunoblotting demonstrated that SH-SY5Y cells transfected with Sw-APP mutant (Sw-APP) had increased pAMPK than SH-SY5Y cells transfected with wt-APP (APP) which had increased pAMPK than control cells transfected with empty-vector (vector). Expression of APPL1 was indifferent among the three groups of cells. Experiments were repeated 3 times with consistent results.

As expected, Western blot analysis of cell lysates revealed that Sw-APP transfected SH-SY5Y cells had higher level of intracellular Aβ oligomers than empty-vector and wt-APP transfected SH-SY5Y cells (p<0.05) (Figure 2A and 2B). ELISA revealed that Sw-APP transfected cells had higher concentration of secreted Aβ oligomers in medium than empty-vector and wt-APP transfected cells (Figure 2C). Western blot analysis also revealed that Sw-APP transfected cells had higher level of pAMPK than wt-APP transfected cells (p = 0.0002), which had higher level of pAMPK than empty-vector transfected control cells (p = 0.0002) whereas APPL1 expression was indifferent between the 3 groups of cells (Figure 3). This may suggest that pAMPK is important for survival of SH-SY5Y cells in the presence of Aβ neurotoxicity, hence Sw-APP and wt-APP transfected cells increased AMPK activation as a compensatory response for survival, especially with abnormal intracellular Aβ accumulation in Sw-APP transfected cells.

### Human Neuroblastoma Cells Transfected with Sw-APP Mutant were more Vulnerable to Cytotoxicity Under Oxidative Stress

**Figure 4:**
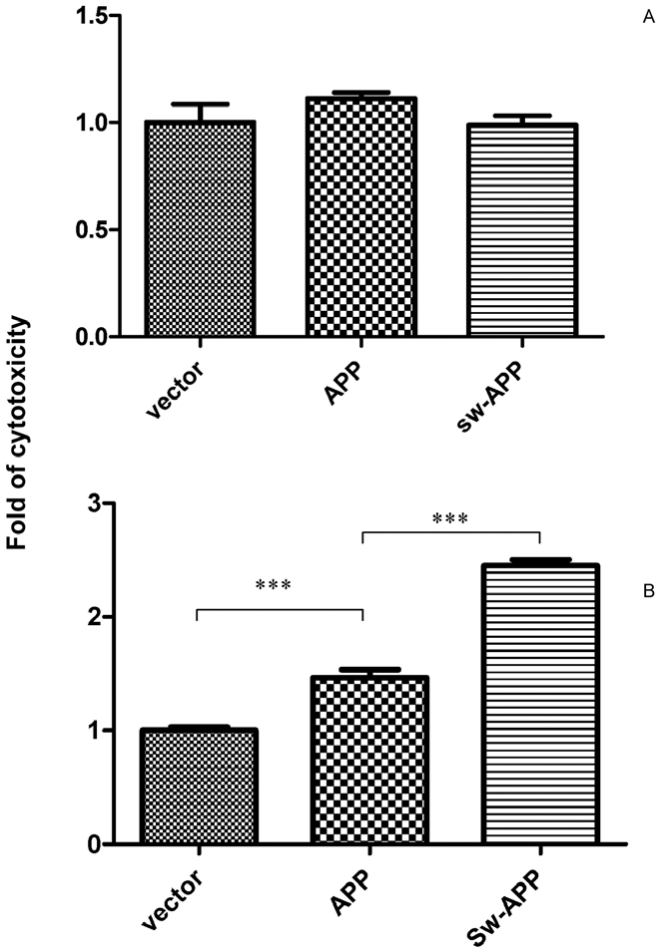
Cytotoxicity of SH-SY5Ycells transfected with empty vector, wild-type APP and the Swedish-APP mutant. **A.** LDH assay revealed that the basal cytotoxicity of Sw-APP transfected (sw-APP), wt-APP transfected (APP) and empty-vector transfected (vector) SH-SY5Ycells are indifferent. **B**. Sw-APP transfected (Sw-APP) cells had increased cytotoxicity under oxidative stress induced by hydrogen peroxide (500 µM) than wt-APP transfected (APP) (p<0.0001) which had increased cytotoxicity under oxidative stress than empty-vector transfected (vector) cells (p<0.0001). Results shown were mean values from 3 independent cultures, and compared by one-way ANOVA using Newman-Keuls multiple comparison test.

Without addition of H_2_O_2_, the basal cytotoxicity/death rates of Sw-APP transfected, wt-APP transfected and empty-vector transfected SH-SY5Ycells were 0.807%, 0.863% and 0.848% respectively, being indifferent (Figure 4A). Upon exposure to H_2_O_2_ induced oxidative stress, Sw-APP transfected SH-SY5Ycells had higher cytotoxicity compared to wt-APP transfected cells (p<0.0001) which had higher cytotoxicity compared to empty-vector transfected cells (p<0.0001) (Figure 4B). This supports that increased Aβ production particularly with abnormal intracellular Aβ accumulation enhances vulnerability to cytotoxicity with cell lysis under oxidative stress [45].

### Adiponectin was Protective against Aβ Induced Neuronal Cytotoxicity Under Oxidative Stress

**Figure 5:**
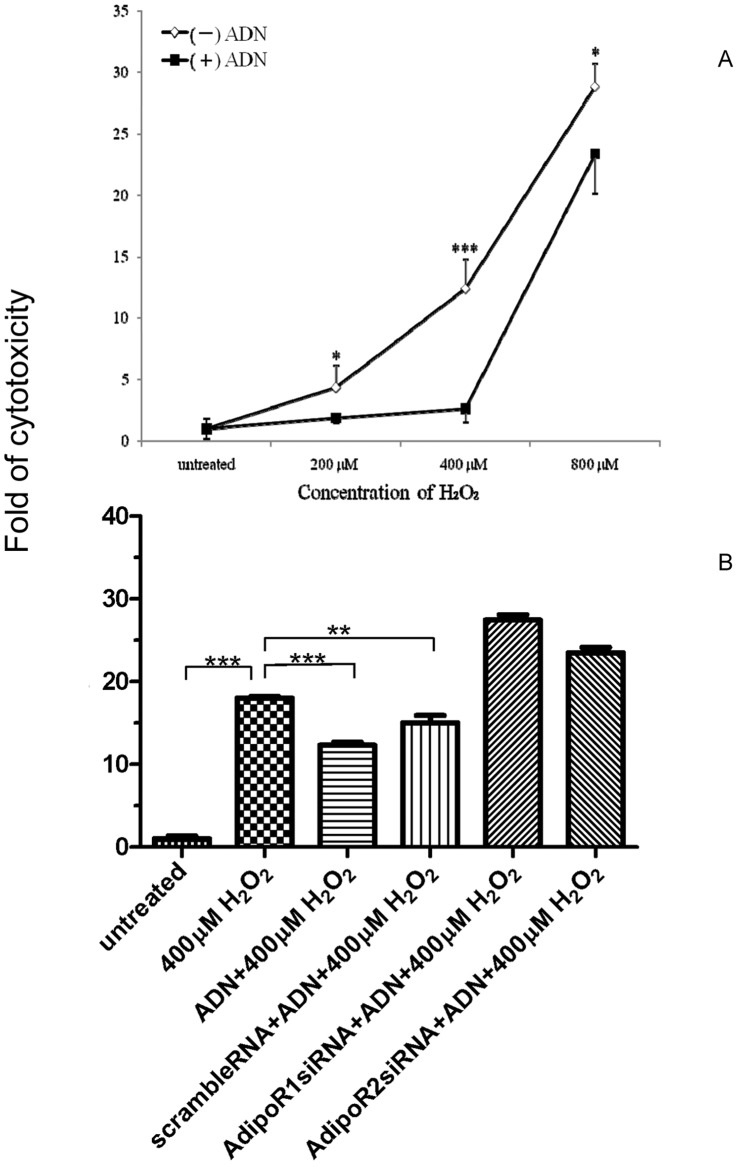
Adiponectin protection against cytotoxicity of SH-SY5Y cells transfected with Swedish-APP (Sw-APP) mutant under oxidative stress. (**A**) Percentage cytotoxicity of SH-SY5Y cells transfected with Sw-APP mutant under oxidase stress by different concentrations of hydrogen peroxide (H_2_O_2_) without and with pretreatment of adiponectin at 10 µg/ml expressed as fold of cytotoxicity relative to cytotoxicity without exposure to H_2_O_2_ (untreated, fold = 1) were shown on y-axis. In all three H_2_O_2_ concentrations, percentage cytotoxicity were significantly reduced with adiponectin pretreatment (p<0.05 for 200 µM H_2_O_2_, p<0.0001 for 400 µM H_2_O_2_ and p<0.05 for 800 µM H_2_O_2_). (−) ADN = without adiponectin pretreatment, (+) ADN = with adiponectin pretreatment. Results shown were mean values from 5 independent cultures, and compared by one-way ANOVA using Newman-Keuls multiple comparison test. (**B**) Using Sw-APP transfected SH-SY5Y cells with knockdown of AdipoR1 or AdipoR2 expression for the cytotoxicity experiments, it was observed that the protective effect of adiponectin against cytotoxicity under oxidative stress was preserved in control Sw-APP transfected SH-SY5Y cells without knockdown of AdipoR1 or AdipoR2 (scramble RNA+ADN+400 µM H_2_O_2_), but lost in cells with knockdown of AdipoR1 (AdipoR1 siRNA+ADN+400 µM H_2_O_2_) or AdipoR2 (AdipoR2 siRNA+ADN+400 µM H_2_O_2_) expression. This confirms that protective effect of adiponectin against cytotoxicity under oxidative stress in Sw-APP transfected SH-SY5Y cells required binding of adiponectin to its cell surface receptors AdipoR1 or AdipoR2. untreated = Sw-APP transfected SH-SY5Y cells without exposure to H_2_O_2_, 400 µM H_2_O_2_ = Sw-APP transfected SH-SY5Y cells exposed to 400 µM H_2_O_2,_ ADN+400 µM H_2_O_2_ = Sw-APP transfected SH-SY5Y cells pretreated with adiponectin before exposure to 400 µM H_2_O_2._ Results shown were mean values from 3 independent cultures, and compared by one-way ANOVA using Newman-Keuls multiple comparison test.

Importantly, pretreatment of Sw-APP transfected SH-SY5Y cells with adiponectin at 10 µg/ml for 2 hours before exposure to H_2_O_2_ led to significant reduction of cytotoxicity under oxidative stress induced by H_2_O_2_ at concentration of 200 µM to 800 µM (Figure 5). Adiponectin of 10 µg/ml is at physiological concentration as documented previously [79]. The most marked protective effect was noted at 400 µM H_2_O_2_ as adiponectin pretreatment lowered cytotoxicity from 12.4 folds to 2.7 folds (p<0.0001). This strongly supports that adiponectin at physiological concentration of 10 µg/ml protects SH-SY5Y cells with Aβ neurotoxicity against cytotoxicity under oxidative stress. To ensure that adiponectin protection against cytotoxicity under oxidative stress induced by H_2_O_2_ in Sw-APP transfected SH-SY5Y cells was due to the effect of adiponectin binding to its cell surface receptors AdipoR1 and AdipoR2, we performed experiments using Sw-APP transfected SH-SY5Y cells with knockdown of AdipoR1 and AdipoR2 expression by RNA interference. The results showed that the protective effect of adiponectin against cytotoxicity under oxidative stress was lost in Sw-APP transfected SH-SY5Y cells with knockdown of AdipoR1 or AdipoR2, but preserved in control Sw-APP transfected SH-SY5Y cells without knockdown of either receptor (scramble RNA) (Figure 5B). This confirms that protective effect of adiponectin against Aβ induced neuronal cytotoxicity under oxidative stress requires binding of adiponectin to its cell surface receptors. With this, the possibility that adiponectin may directly interact with Aβ oligomers leading to blockade of Aβ oligomers-associated cytotoxic effects is unlikely.

### Adiponectin Protection against Aβ Induced Neuronal Cytotoxicity Under Oxidative Stress Involved APPL1-dependent AMPK Activation

**Figure 6:**
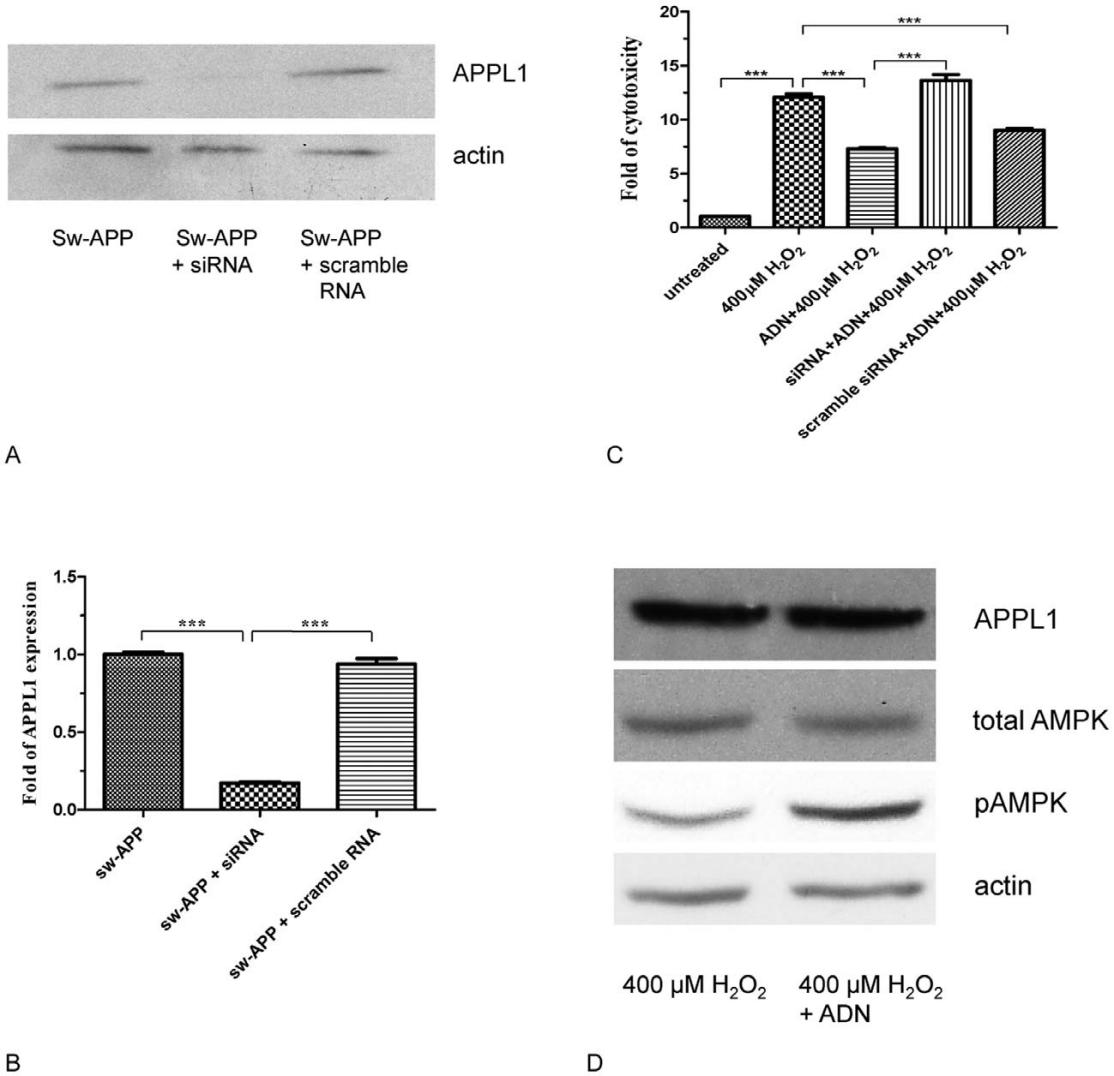
Adiponectin neuroprotection against cytotoxicity under oxidative stress is mediated via APPL1-dependent AMPK activation. **A**. Western blot analysis of control Swedish-APP mutant transfected SH-SY5Y cells (Sw-APP) and similar cells transfected with scramble RNA (Sw-APP+scramble RNA) revealed APPL1 expression. Successful knowkdown of APPL1 expression by RNA interference was shown on immunoblotting of cell lysate of Sw-APP transfected SH-SY5Y cells transfected with short interference RNA for APPL1 (Sw-APP+siRNA). **B**. Image-J analysis confirmed successful knockdown of APPL1 expression in Sw-APP transfected SH-SY5Y cells by siRNA compared to control cells transfected with scramble RNA (p<0.0001). Transfection experiments were performed 3 times with consistent results. **C**. Neuroprotective effect of adiponectin against cytotoxicity under oxidative stress (400 µM H_2_O_2_) was lost in Sw-APP transfected SH-SY5Y cells with knockdown of APPL1 expression (siRNA+ADN+400 µM H_2_O_2_), but preserved in cells without knockdown of APPL1 expression (scramble siRNA+AND+400 µM H_2_O_2_, p<0.0001). This suggests that adiponectin neuroprotection against Aβ-induced cytotoxicity under oxidative stress requires APPL1. Fold of percentage cytotoxicity relative to cells not exposed to H_2_O_2_ (untreated, fold = 1) was shown on y-axis. untreated = Sw-APP transfected SH-SY5Y cells without exposure to H_2_O_2_, 400 µM H_2_O_2_ = Sw-APP transfected SH-SY5Y cells exposed to 400 µM H_2_O_2,_ ADN+400 µM H_2_O_2_ = Sw-APP transfected SH-SY5Y cells pretreated with adiponectin before exposure to 400 µM H_2_O_2._ Results shown were mean values from 3 independent cultures, and compared by one-way ANOVA using Newman-Keuls multiple comparison test. **D**. Adiponectin induced AMPK activation. Western blot analysis of cell lysates from Sw-APP transfected SH-SY5Y cells exposed to oxidative stress (400 µM H_2_O_2_) demonstrated that cells pretreated with adiponectin (ADN) had increased phosphorylated AMPK (pAMPK) compared to cells without adiponectin pretreatment whereas APPL1 and total AMPK levels were similar. This strongly suggests that adiponectin neuroprotection against cytotoxicity under oxidase stress in Sw-APP transfected SH-SY5Y cells is mediated via APPL1-dependent AMPK activation. Western blot analysis were performed for 3 independent cultures with consistent results.

We further investigated the mechanisms of neuroprotection by adiponectin against Aβ neurotoxicity. As important cellular functions of adiponectin are exerted via APPL1-mediated AMPK activation leading to increased intracellular pAMPK, we proceeded to study whether protective effect of adiponectin against Aβ neurotoxicity involved APPL1-dependent AMPK activation. Using Sw-APP transfected SH-SY5Y cells with knockdown of APPL1 expression by RNA interference (Figures 6A and 6B), we found that the neuroprotective effect of adiponectin against cytotoxicity under H_2_O_2_ induced oxidative stress was lost in cells with knockdown of APP1 expression but preserved in cells without knockdown of APPL1 expression (Figure 6C). These suggest that neuroprotective effect of adiponectin against Aβ mediated neuronal cytotoxicity under oxidative stress is dependent on APPL1. In addition, Western blot analysis of cell lysates revealed that Sw-APP transfected SH-SY5Y cells pretreated with adiponectin before exposure to H_2_O_2_ had higher pAMPK level than Sw-APP transfected SH-SY5Y cells without adiponectin pretreatment_,_ while total AMPK level was similar (Figure 6D). These together suggest that the protective effect of adiponectin against Aβ induced neuronal cytotoxicity under oxidative stress is mediated via APPL1-dependent AMPK activation.

### Human Neuroblastoma Cells Transfected with Sw-APP Mutant and wt-APP had Increased NF-κB Activation

**Figure 7:**
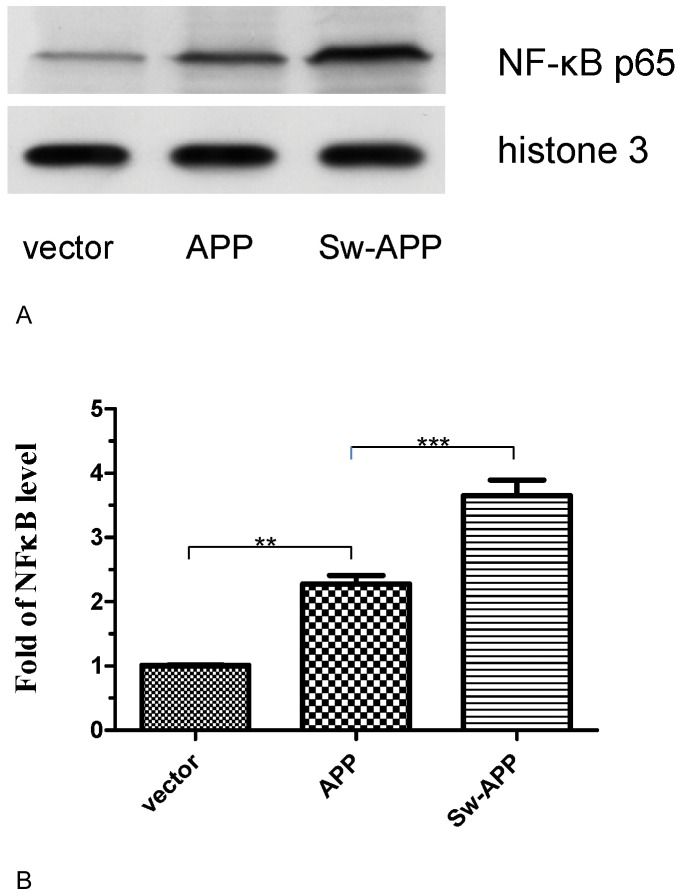
Western blot for nuclear factor-kappa B (NF-κB) activation. **A**. SH-SY5Ycells transfected with the Swedish-APP mutant (Sw-APP) had significantly higher level of nuclear NF-κB p65 than cells transfected with wild-type APP (APP), which had higher level of nuclear NF-κB p65 than cells transfected with empty-vector (vector). The control nuclear protein was histone 3. **B** Image J and statistical analysis confirmed statistically significant difference in the level of NF-κB activation. Sw-APP transfected SH-SY5Ycells under greatest Aβ neurotoxicity had the highest level of nuclear NF-κB p65 more than that of wt-APP transfected cells (p<0.0001), which had higher level of NF-κB p65 than empty-vector transfected cells without Aβ neurotoxicity (p<0.001). Results shown were mean values from 3 independent cultures, and compared by one-way ANOVA using Newman-Keuls multiple comparison test.

Western blot analysis of nuclear extract for p65 component of NF-κB (NF-κB p65) revealed that Sw-APP transfected SH-SY5Y cells had higher NF-κB p65 level than wt-APP transfected cells (p<0.0001), which had higher NF-κB p65 level than empty-vector transfected cells (p<0.001) (Figure 7). These suggest that Aβ over-production and Aβ neurotoxicity lead to increased NF-κB activation, consistent with the concept that neuroinflammation is one of the pathogenetic mechanisms in AD.

### NF-κB Activation was Reduced in Sw-APP Transfected Human Neuroblastoma Cells Pretreated with Adiponectin

**Figure 8:**
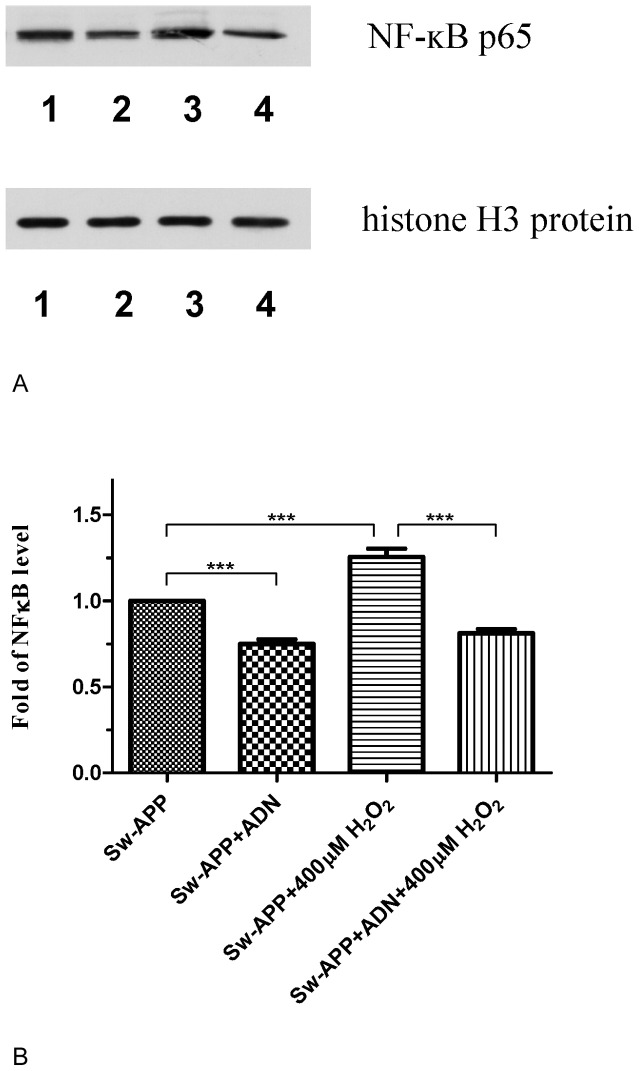
Adiponectin (ADN) suppressed NF-κB activation. **A**. Western blot of nuclear extracts revealed that SH-SY5Y cells transfected with Swedish-APP mutant had high level of NF-κB p65 signifying NF-κB activation (lane 1), and treatment with adiponectin (ADN) at 10 µg/ml for 2 hours resulted in reduced NF-κB activation (lane 2). Sw-APP transfected cells exposed to 400 µM H_2_O_2_ for 2 hours had high level of NF-κB activation (lane 3), and importantly Sw-APP transfected cells pretreated with adiponectin at 10 µg/ml for 2 hours before exposure to 400 µM H_2_O_2_ had reduced NF-κB activation (lane 4). Control nuclear protein was histone H3. **B**. Statistical analysis of imaging J values revealed that Sw-APP transfected SH-SY5Y cells (Sw-APP) had significant reduction level of NF-κB activation after treatment with ADN (Sw-APP+ADN, p<0.0005). Consistently, Sw-APP transfected SH-SY5Y cells exposed to oxidative stress of 400 µM H_2_O_2_ (Sw-APP+400 µM H_2_O_2_) had significantly reduced level of NF-κB activation with adiponectin pretreatment at 10 µg/ml for 2 hours before exposure to oxidative stress of 400 µM H_2_O_2_ (Sw-APP+AND+400 µM H_2_O_2_, p<0.0005). This raises the possibility that one mechanism underlying adiponectin neuroprotection against Aβ neurotoxicity-induced cytotoxicity under oxidative stress is suppression of NF-κB activation. Results shown were mean values from 3 independent cultures, and compared by one-way ANOVA using Newman-Keuls multiple comparison test.

We further studied whether adiponectin might suppress NF-κB activation especially adiponectin is known to possess anti-inflammatory action. Western blot analysis of nuclear fraction of Sw-APP transfected SH-SY5Y cells revealed that cells treated with adiponectin at 10 ug/ml for 2 hours had significantly reduced NF-κB activation compared to untreated cells (p<0.0001) (Figure 8A and 8B). Similar reduction of NF-κB activation in Sw-APP transfected SH-SY5Y cells exposed to H_2_O_2_ induced oxidative stress cells was observed with adiponectin pretreatment before exposure to H_2_O_2_ (p<0.0001) (Figure 8A and 8B). These raise the possibility that one of the mechanisms underlying adiponectin protection against Aβ neurotoxicity was suppression of NF-κB activation, hence attenuating neuroinflammation.

## Discussion

Aβ inhibits enzymes of mitochondrial oxidative phosphorylation and key enzymes of Krebs cycle, leading to mitochondrial dysfunction, reduced ATP production (energy failure), and generation of ROS and reactive nitrogen species (RNS) such as superoxides and nitrites. ROS and RNS contribute to oxidative stress which causes oxidation and nitration of proteins, nucleic acids and lipids leading to mitochondrial dysfunction, altered Ca^2+^ homeostasis, membrane damage and altered proteasome function with abnormal protein accumulation [49]. ROS also activate genes involved in inflammatory pathways such as IL-6, interferon-gamma (IFN-γ) and inducible nitric oxide synthase (iNOS) [17]. Activation of iNOS leads to excess endogenous nitric oxide (NO) which causes S-nitrosylation of dynamin-related protein-1, resulting in mitochondrial fission, synaptic loss and neuronal damage [50]. Hence, oxidative stress causes cellular dysfunctions, neuronal degeneration and death [49], [51]. Importantly, increased oxidative stress occurs early in AD [51]–[53].

We demonstrated that Sw-APP transfected human neuroblastoma cells over-producing Aβ had increased susceptibility to cytotoxicity under oxidative stress. This supports that Aβ neurotoxicity mediated oxidative stress and mitochondrial dysfunction play important roles in AD pathogenesis [26], [49], [51]. The Sw-APP transfected SH-SY5Y cells had higher secreted Aβ oligomers level in culture medium and intracellular Aβ oligomers level compared to empty-vector transfected cells. We are uncertain whether the increased susceptibility to cytotoxicity under oxidative stress is due to the extracellular or intracellular Aβ oligomers, but probably the latter. Our observation that AMPK activation was increased in Sw-APP transfected cells compared to wt-APP and empty-vector transfected cells may imply that increased pAMPK level from AMPK activation is beneficial for neuronal survival under Aβ neurotoxicity. AMPK is a sensor of cellular energy level and is activated by increase in AMP to ATP ratio, hence functions to preserve cellular energy. AMPK activation upregulates ATP-producing catabolic pathways such as uptake and metabolism of glucose and fatty acids, and downregulates ATP-consuming anabolic pathways such as synthesis of glycogen, cholesterol and proteins [54]. It is likely that Aβ neurotoxicity in the Sw-APP transfected neuroblastoma cells induces mitochondrial dysfunction leading to decreased ATP level, hence stimulates AMPK activation. Treatment of cultured embryonic rat hippocampal neurons under glucose deprivation with a potent AMPK activator led to improved cell survival. Similar beneficial effect was also found in neurons exposed to Aβ peptide, sodium cyanide (mitochondrial toxin) and glutamate [55]. However, Thornton et al. reported that treatment of primary mouse cortical neurons with Aβ42 induces AMPK activation which leads to increased tau phosphorylation. This pathway of Aβ42 induced AMPK activation involves the N-methyl-D-aspartate (NMDA) receptor as AMPK activation is inhibited by memantine, a partial NMDA receptor antagonist used in treatment of AD [56]. Consistently, Vingtdeux et al. reported that activated AMPK (pAMPK) is abnormally accumulated in cerebral neurons of patients with tauopathies including AD. In AD, pAMPK is accumulated in neuropil threads and dystrophic neurites surrounding amyloid plaques in >90% of neurons bearing neurofibrillary tangles and pre-tangles. The investigators proposed that AMPK might regulate neurodegeneration by controlling tau phosphorylation [57]. Whether AMPK activation leads to tau phosphorylation cannot be addressed in our study.

Une at al. reported that plasma adiponectin level was significantly higher in mild cognitive impairment (MCI) and AD patients than normal controls (NC), whereas CSF adiponectin level was significantly higher in MCI than NC [58]. This increased adiponectin level may be a compensatory response to protect against neurodegeneration in MCI or early AD. However, van Himbergen et al. studied 840 dementia-free Framingham Heart Study participants over a mean follow-up period of 13 years and reported that 159 persons developed dementia (125 AD); and after adjustment of other risk factors, only adiponectin in women was associated with an increased risk of all-cause dementia (hazard ratio [HR] 1.29, p = 0.054) and AD (HR 1.44, p = 0.050) per 1 standard deviation increase in adiponectin level. In addition, women with baseline adiponectin levels above the median had a higher risk of all-cause dementia (HR 1.63, p = 0.04) and AD (HR 1.87, p = 0.01) compared to those with adiponectin levels below median [59]. The exact role of adiponectin in human cognitive functions awaits further clarification. Importantly, our results demonstrated that adiponectin at a physiological concentration of 10 µg/ml (physiological serum concentration 3–30 µg/ml) is protective against cytotoxicity of SH-SY5Y cells expressing Sw-APP mutant under H_2_O_2_ induced oxidative stress; and this protective effect of adiponectin against Aβ neurotoxicity is dependent on APPL1, likely mediated via APPL1-dependent AMPK activation. APPL1 plays important role as an endosomal adaptor protein in various cellular signaling pathways [38], [60]. AMPK activation promotes ATP producing catabolic pathways including glucose uptake and metabolism which is diminished in AD brain with insulin deficiency and resistance [21]. AMPK activation protects and confers survival benefits to neurons against oxidative, metabolic and excitotoxic stress [54]–[55]. Resveratrol, a neuroprotective plant polyphenol, promotes neurite outgrowth and mitochondrial biogenesis in neurons via AMPK activation [61]. In addition, adiponectin-mediated activation of AMPK, p38MAPK and Rab5 leads to increased glucose transporter 4 (Glut4) membrane translocation [62] which also promotes neuronal glucose uptake and metabolism. Insulin is important for neuronal function and survival, and reduced cerebral insulin and insulin-like growth factor (IGF-1) levels, as well as impaired neuronal insulin signaling are noted in AD brain [63]–[64]. Both the expression and function of insulin and IGF-1 deteriorate with progression of AD [57], and intranasal insulin improves cognition and modulates Aβ in early AD [65]. The insulin-sensitizing action of adiponectin [33], [60] may be another mechanism of neuroprotection in AD which cannot be addressed in our study.

Neuroinflammation is increasingly recognized as an important element in AD [22], [24], [66]–[67]. Reactive astrocytes in close proximity to Aβ plaques and activated microglia secrete inflammatory mediators including IL-1β, TNF-α, IL-6, interleukin-18 (IL-18) and iNOS which generate free radicals such as NO. In addition, Aβ undergoes non-enzymatic glycation to form advanced glycation endproducts (AGEs) which bind to receptor for AGES (RAGE). This AGES/RAGE signaling activity upregulates activity of the transcription factor, nuclear factor kappa-B (NF-κB) [68]–[70]. NF-κB activation is a central event of neuroinflammation in AD [71], and is predominantly found in neurons and glial cells at regions surrounding Aβ plaques in AD brain [72]–[75]. NF-κB activation triggers expression of pro-inflammatory molecules including cytokines and chemokines which contribute to neuronal cytotoxicity in AD [71]. This is consistent with our observation that Sw-APP transfected and wt-APP transfected neuroblastoma cells overproducing Aβ have increased NF-κB activation than empty-vector transfected cells not overproducing Aβ. Importantly, NF-κB activation in Sw-APP transfected neuroblastoma cells diminished with adiponectin treatment in parallel with its protective effect against cytotoxicity under oxidative stress. This raises the possibility that suppression of NF-κB activation is one mechanism underlying neuroprotective effect of adiponectin against Aβ-induced neuronal cytotoxicity under oxidative stress. Adiponection was recently reported to block IL-18 mediated endothelial cell death [76]. Treatment of endothelial cells with IL-18 suppressed Akt (protein kinase B) phosphorylation and its associated kinase activity [77], and induced IκB kinase (IKK)-NF-κB-dependent phosphatase and tensin homolog (PTEN) activation, hence promoted endothelial cell death [76]. Pretreatment with adiponectin stimulated APPL1-dependent AMPK activation, reversed Akt inhibition, inhibited IKK-NF-κB dependent PTEN expression in an AMPK-dependent manner, hence blocked IL-18 mediated endothelial cell death [76]. This is consistent with the cytoprotective effect of adiponection via AMPK activation which inhibits IKK-NF-κB dependent-PTEN pathway [60]. IL-18 is upregulated in AD brain [78] and IL-18 production by peripheral blood cells is increased in AD patients, the level of which correlates with cognitive impairment [79]. These suggest that IL-18 related inflammatory pathways are exacerbated in AD, which can be attenuated by adiponectin. In addition, adiponectin was reported to protect neurons against ischemic-reperfusion injury in a rat model of stroke through anti-inflammatory action via inhibition of NF-κB activation [80].

This is the first report that adiponectin is protective in human neuroblastoma cells with Aβ neurotoxicity. Adiponectin has been reported to be neuroprotective in 1) SH-SY5Y cells against MPP^+^-induced cytotoxicity [81], 2) SH-SY5Y cells against acetaldehyde-induced apoptosis [48], 3) a mouse model of epilepsy against kainic acid-induced excitotoxicity [82] and 4) a rat model of stroke against cerebral ischemia-reperfusion injury [80]. It is encouraging that a recent study reported that intense expression of AdipoR1 was noted in the hypothalamus and the nucleus basalis of Meynert in basal forebrain which is frequently affected in AD [83]. Further studies are needed to confirm the neuroprotective effect of adiponectin in Aβ neurotoxicity, which bears potential for novel therapies against AD. Interestingly, another adipokine, leptin has been shown to reduce Aβ levels in neuronal cells via AMPK activation [84], and reduce pathology and improve memory in a transgenic mouse AD model [85].
